# Supplementary figures and images for: A conserved regulatory mechanism mediates the convergent evolution of plant shoot lateral organs
Source: PLoS Biol. 2019 Dec 9;17(12):e3000560. doi: 10.1371/journal.pbio.3000560 (PMC6901180; doi:10.1371/journal.pbio.3000560)

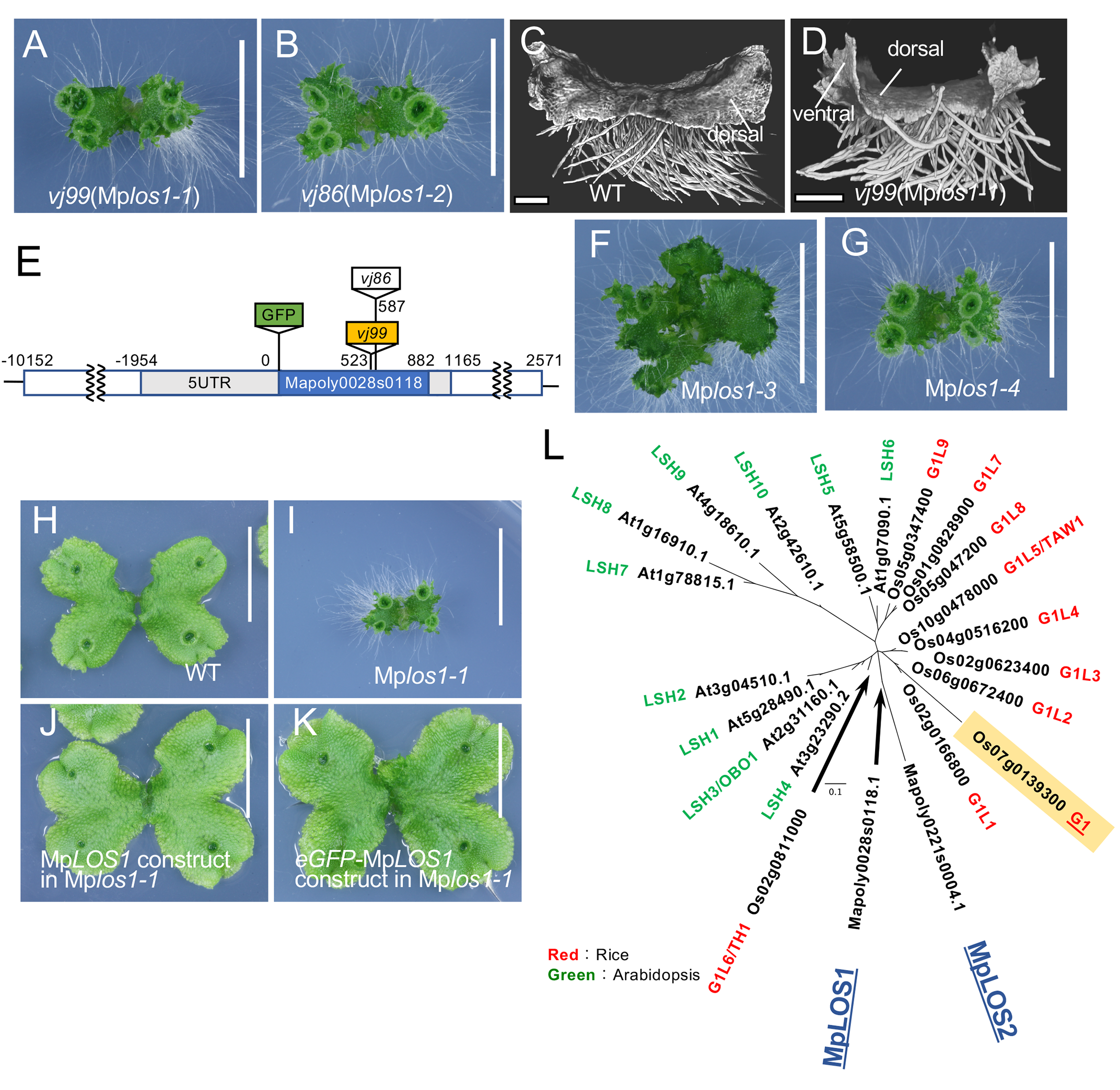

Supplement: S1 Fig — (A and B) Phenotypes of vj99 and vj86 mutants. (C and D) LSFM image of gross morphology of WT (C) and vj99 mutant (D) gemmalings. (E) Overview of the functional MpLOS1 construct and the T-DNA insertion mutants isolated by forward genetic screening. The regions 10,152 bp upstream and 1,689 bp downstream of coding sequences were used to express MpLOS1. (F and G) Phenotypic series of Mplos1 knockout mutants. (H-K) Complementation of Mplos1-1 mutants with a functional MpLOS1 construct. The phenotype of Mplos1-1 (I) is complemented by introducing the genomic MpLOS1 fragment (J) as well as the eGFP-fused MpLOS1 genomic fragment (K). (L) Phylogenetic tree of ALOG family proteins in Arabidopsis, rice, and Marchantia. Green, red, and blue symbols indicate ALOG proteins in Arabidopsis, rice, and Marchantia, respectively. Alignment of ALOG family proteins can be found in S1 Data. Scale bars = 1 cm in (A, B, F, G, H, I, J, and K) and 500 μm in (C and D). ALOG, Arabidopsis LIGHT-DEPENDENT SHORT HYPOCOTYLS 1 and Oryza G1; eGFP, enhanced green fluorescent protein; LSFM, light sheet fluorescence microscopy; MpLOS1, M. polymorpha LATERAL ORGAN SUPRESSOR 1; T-DNA, transfer DNA; WT, wild type. (TIF) [file pbio.3000560.s001.tif]

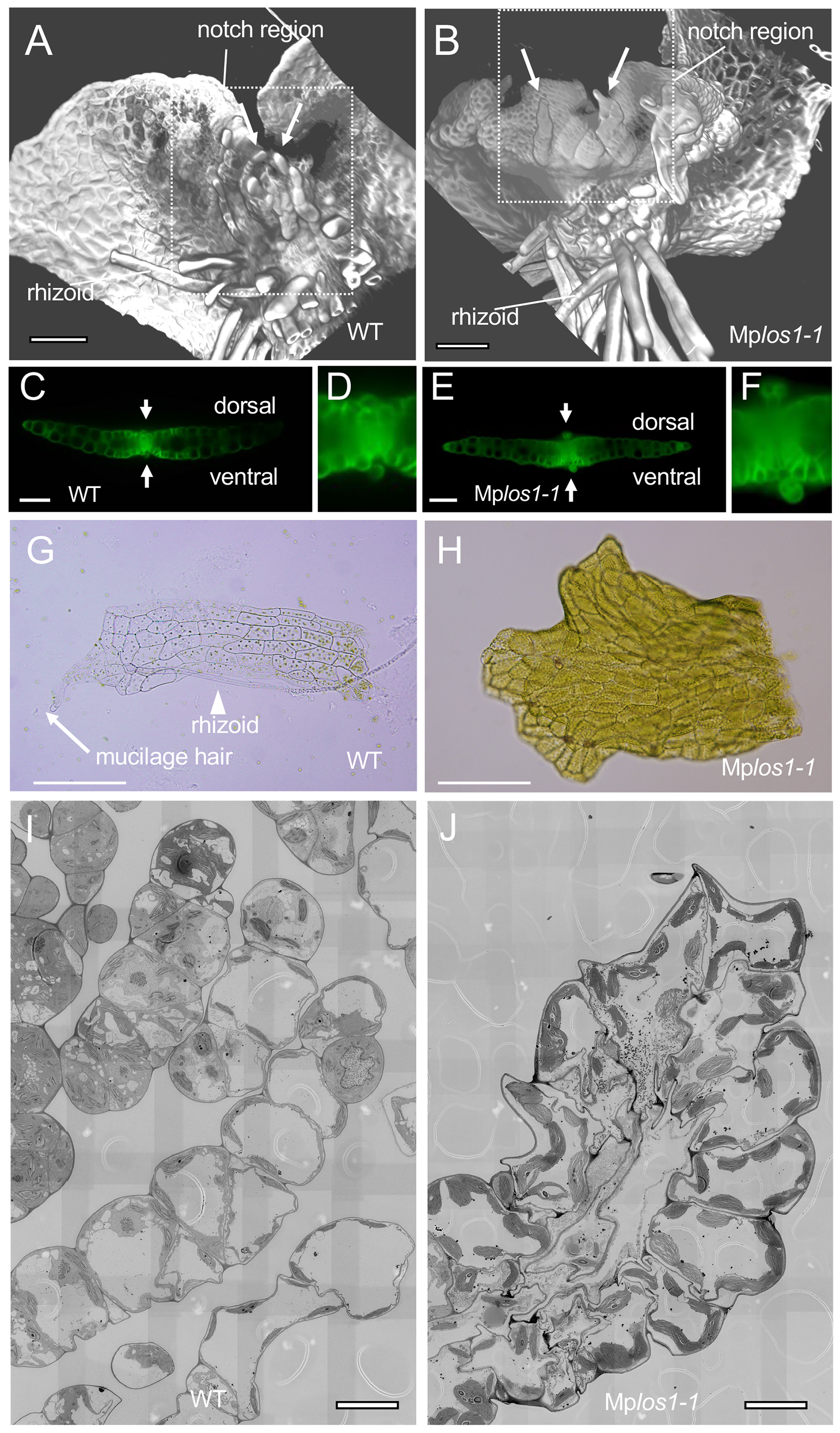

Supplement: S2 Fig — (A and B) LSFM image of gemmalings in WT (A) and in Mplos1-1 mutants (B) observed from the ventral side. Dotted boxes in (A) and (B) that include apical notch regions are shown as close-up images in Fig 2(E) and 2(F), respectively. Ventral scales in WT or their corresponding tissues in Mplos1-1 mutants are indicated by arrows. (C-F) Vertical transverse optical sections of apical notch regions in 4-day-old gemmalings obtained by LSFM. Optical sections in WT (C and D) and in Mplos1-1 mutants (E and F) are shown. Note that the number of cells that make up mucilage and ventral scales increased, and thus, these tissues became larger in Mplos1-1 mutants. (G and H) Images of ventral scales in WT (G) and the corresponding tissues in Mplos1-1 mutants (H). Note that ventral scale cells are transformed into green tissues that lack rhizoids and mucilage hair cells in Mplos1-1 mutants. Rhizoids and mucilage hair in WT are indicated by an arrowhead and an arrow, respectively. (I and J) FESEM images of ventral scale cells in WT (I) and the corresponding cells in Mplos1-1 mutants (J). Images were composites, made up of 25 and 30 individual images (tiles) in WT and Mplos1-1 mutants, respectively. Scale bars = 150 μm in (A and B), 100 μm in (C and E), 200 μm in (G and H), and 10 μm in (I and G). MpLOS1, Marchania polymorpha LATERAL ORGAN SUPRESSOR 1; LSFM, light sheet fluorescence microscopy; FESEM, field emission scanning electron microscopy; WT, wild type. (TIF) [file pbio.3000560.s002.tif]

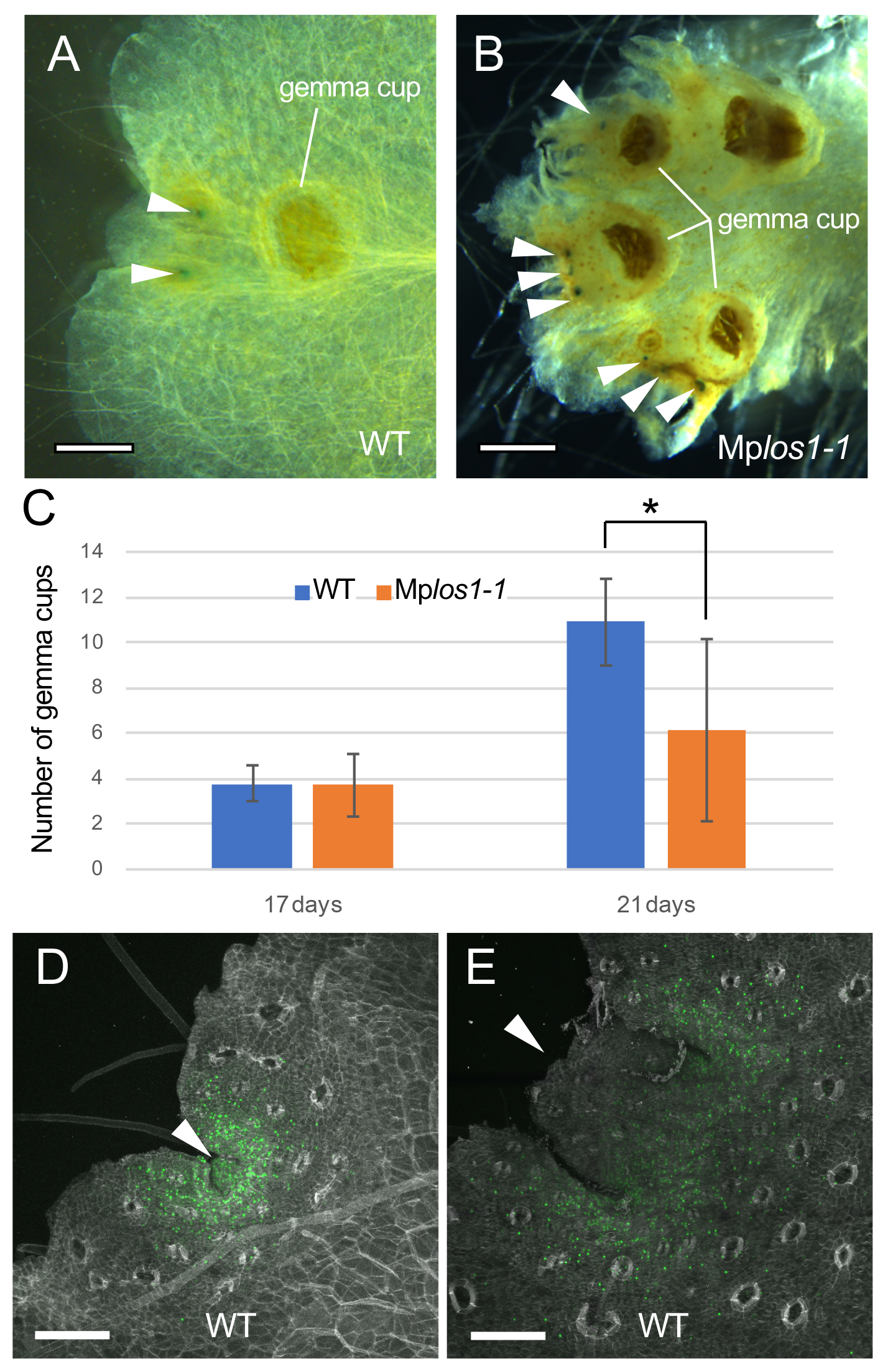

Supplement: S3 Fig — (A and B) Apices stained by proMpYUC2:GUS in 3-week-old gemmalings in WT (A) and Mplos1-1 mutants (B). Arrowheads indicate GUS staining at apical notches. (C) Number of gemma cups in WT Tak1 and in Mplos1-1 mutants. Each bar indicates the mean ± SD. At least seven gemmalings were analyzed at each time point. (D and E) EdU labeling in central lobes. EdU-positive signals of 5-day-old WT gemmalings (D) and that of 9-day-old WT gemmalings (E) are shown. Note that there is little EdU labeling in the central lobes in 9-day-old gemmalings. Arrowhead indicates central lobes. Scale bars = 1.5 mm in (A and B) and 200 μm in (D and E). p-Values lower than 0.01 are indicated by asterisks (*). Underlying data for this figure can be found in S2 Data. EdU, 5-ethynyl-2′-deoxyuridine; GUS, ß-glucuronidase; MpLOS1, M. polymorpha LATERAL ORGAN SUPRESSOR 1; proMpYUC2, promoter M. polymorpha YUCCA2; Tak1, Takaragaike-1; WT, wild type. (TIF) [file pbio.3000560.s003.tif]

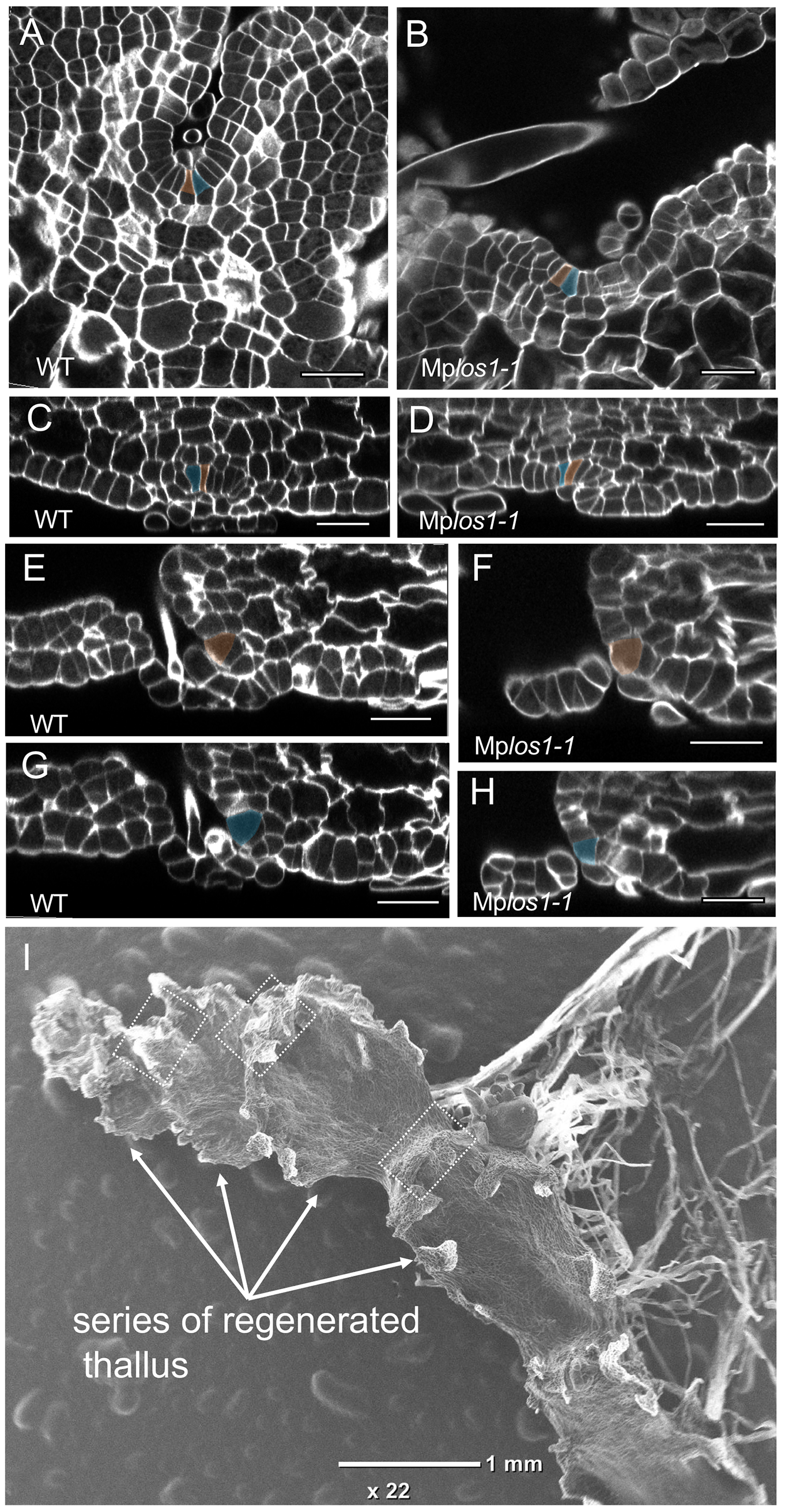

Supplement: S4 Fig — (A-H) Detailed cellular organization of apical notches in 3-day-old gemmalings in WT (A, C, E, and G) and in Mplos1-1 mutants (B, D, F, and H). Horizontal (A and B), vertical transversal (C and D), and vertical longitudinal optical sections (E-H) obtained after the 3D reconstruction of a series of CLSM images were shown. The cells highlighted by orange or cyan in (A) and (B) were sectioned in vertical longitudinal and vertical transversal directions, respectively. The cells highlighted by orange or cyan in (A-H) are identical cells. Cell walls were stained using Direct Red 23. (I) SEM image of Mplos1-1 mutant gemmalings. Thallus regeneration occurs in Mplos1-1 mutants next to aborted meristems. The aborted meristems are indicated by dotted boxes. Scale bars = 30 μm in (A-H) and 1 mm in (I). CLSM, confocal laser scanning microscopy; MpLOS1, M. polymorpha LATERAL ORGAN SUPRESSOR 1; SEM, scanning electron microscope; WT, wild type. (TIF) [file pbio.3000560.s004.tif]

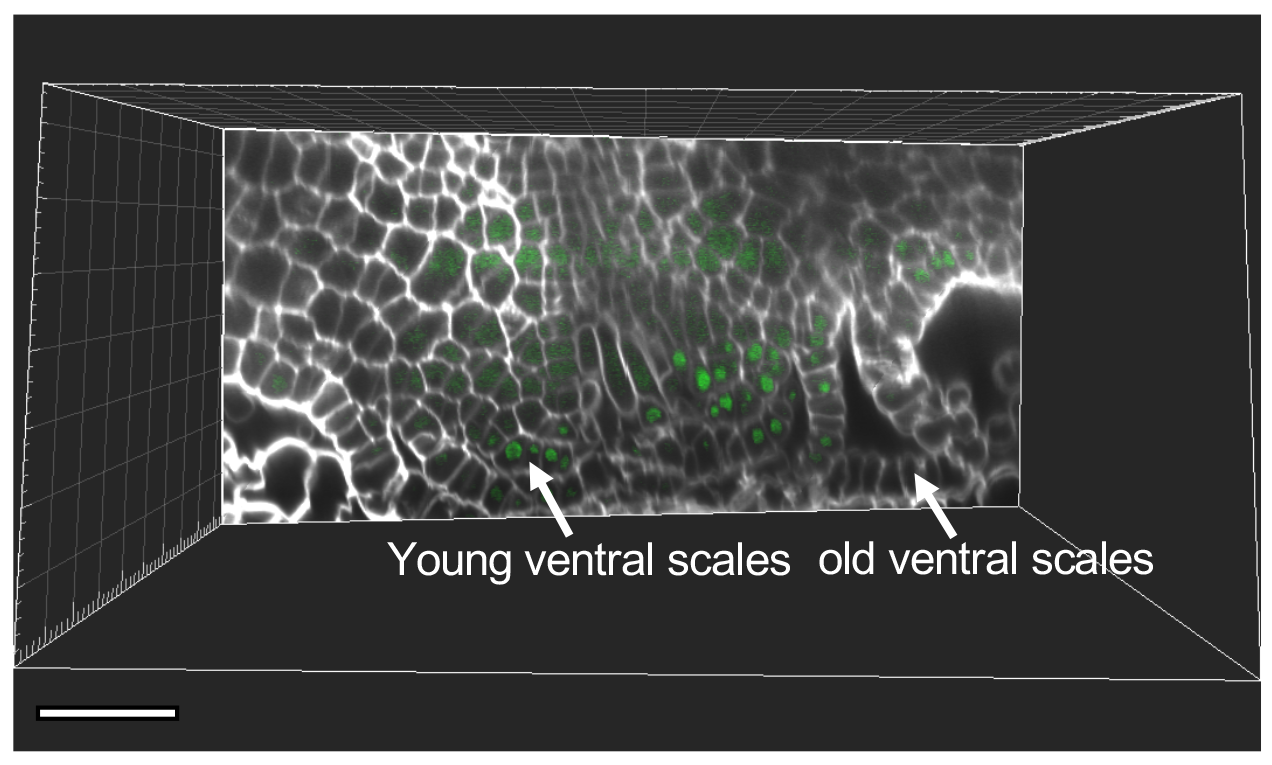

Supplement: S5 Fig — CLSM image of Mplos1-1 mutant gemmalings that express proMpLOS1:eGFP-MpLOS1 constructs. Vertical transverse sections were obtained after the 3D reconstruction of a series of CLSM images. Cell walls were stained by calcofluor. Scale bar = 50 μm. CLSM, confocal laser scanning microscopy; eGFP, enhanced green fluorescent protein; MpLOS1, M. polymorpha LATERAL ORGAN SUPRESSOR 1. (TIF) [file pbio.3000560.s005.tif]

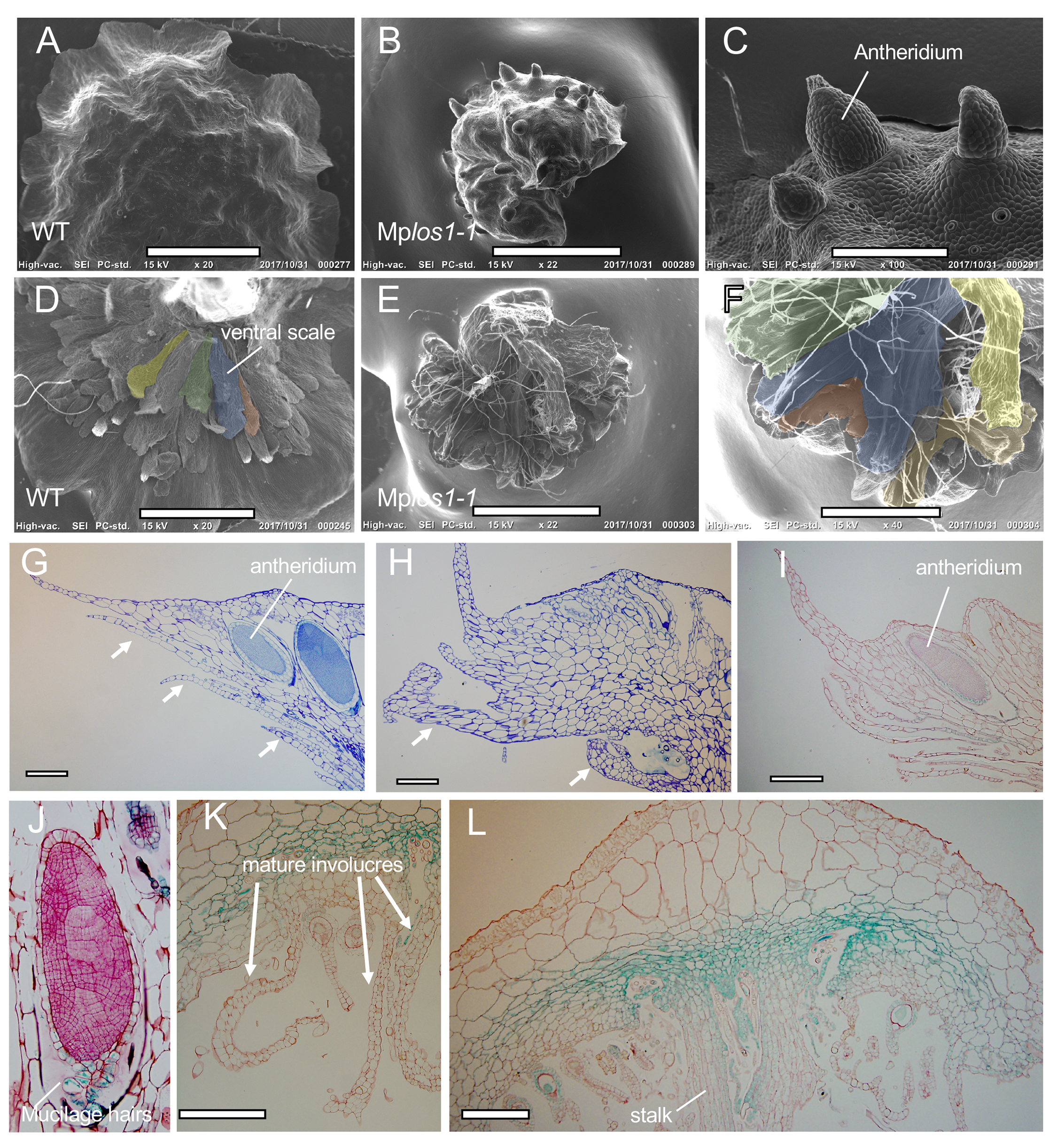

Supplement: S6 Fig — (A-F) SEM image of antheridiophores in WT Tak1 (A and D) and Mplos1-1 mutants (B, C, E, and F). The dorsal side (A-C) and ventral side (D-F) of antheridiophores are shown. (C) and (F) are close-up images of (B) and (E), respectively. Some ventral scales are highlighted by colors. Note the exaggerated growth of ventral scales as compared with the size of thalli (F). (G and H) Vertical sections of antheridia in WT (G) and Mplos1-1 mutants (H). Note the extra cell division of misspecified ventral scales in Mplos1-1 mutants. Ventral scales (G) and misspecified ventral scales (H) are indicated by arrows. (I and J) Cross sections of GUS-stained antheridiophores (I) and antheridia (J) that express proMpLOS1:GUS constructs. Note that GUS activities were detected in the ventral scales in WT (I). (K and L) Cross sections of GUS-stained archegoniophores that express proMpLOS1:GUS. Regions that include mature involucres (K) and the whole image of archegoniophores (L) are shown. Note that in contrast to immature archegoniophores, GUS activity was not detected in mature involucres. Scale bars = 2 mm in (A, B, and E), 1 mm in (F), 400 μm in (C), and 200 μm in (G, H, I, K, and L). GUS, ß-glucuronidase; MpLOS1, M. polymorpha LATERAL ORGAN SUPRESSOR 1; SEM, scanning electron microscope; Tak1, Takaragaike-1; WT, wild type. (TIF) [file pbio.3000560.s006.tif]
