# Supplementary material for: A conserved regulatory mechanism mediates the convergent evolution of plant shoot lateral organs
Source: PLoS Biol. 2019 Dec 9;17(12):e3000560. doi: 10.1371/journal.pbio.3000560 (PMC6901180; doi:10.1371/journal.pbio.3000560)
Supplement: S1 MDAR Checklist — MDAR, Materials Design Analysis Reporting. (DOCX) [file pbio.3000560.s010.docx]

Materials Design Analysis Reporting (MDAR)

Checklist for Authors

The MDAR framework establishes a minimum set of requirements in transparent reporting applicable to studies in the life sciences (see Statement of Task: doi:10.31222/osf.io/9sm4x.). The MDAR checklist is a tool for authors, editors and others seeking to adopt the MDAR framework for transparent reporting in manuscripts and other outputs. Please refer to the MDAR Elaboration Document for additional context for the MDAR framework.

Materials

| **Antibodies** | **Yes (indicate where provided: page no/section/legend)** | **n/a** |
| --- | --- | --- |
| For commercial reagents, provide supplier name, catalogue number and RRID, if available. |  | n/a |
|  |  |  |
| **Cell materials** | **Yes (indicate where provided: page no/section/legend)** | **n/a** |
| **Cell lines:** Provide species information, strain. Provide accession number in repository **OR** supplier name, catalog number, clone number, **OR** RRID |  | n/a |
| **Primary cultures:** Provide species, strain, sex of origin, genetic modification status. |  | n/a |
|  |  |  |
| **Experimental animals** | **Yes (indicate where provided: page no/section/legend)** | **n/a** |
| **Laboratory animals:** Provide species, strain, sex, age, genetic modification status. Provide accession number in repository **OR** supplier name, catalog number, clone number, **OR** RRID |  | n/a |
| **Animal observed in or captured from the field:** Provide species, sex and age where possible |  | n/a |
| **Model organisms:** Provide Accession number in repository (where relevant) **OR** RRID |  | n/a |
|  |  |  |
| **Plants and microbes** | **Yes (indicate where provided: page no/section/legend)** | **n/a** |
| **Plants:** provide species and strain, unique accession number if available, and source (including location for collected wild specimens) | Marchantia polymorpha Tak1 and Tak2 strains were used in this manuscript. These materials are used in Figure1-5.  Oryza sativa, Nipponbare strain is used in this manuscript. This material is used in Figure6.  Marchantia and Oryza strains are described in the section of materials and methods. |  |
| **Microbes:** provide species and strain, unique accession number if available, and source |  | n/a |
|  |  |  |
| **Human research participants** | **Yes (indicate where provided: page no/section/legend)** | **n/a** |
| Identify authority granting ethics approval (IRB or equivalent committee(s), provide reference number for approval. |  | n/a |
| Provide statement confirming informed consent obtained from study participants. |  | n/a |
| Report on age and sex for all study participants. |  | n/a |

Design

| **Study protocol** | **Yes (indicate where provided: page no/section/legend)** | **n/a** |
| --- | --- | --- |
| For clinical trials, provide the trial registration number **OR** cite DOI in manuscript. |  | n/a |
|  |  |  |
| **Laboratory protocol** | **Yes (indicate where provided: page no/section/legend)** | **n/a** |
| Provide DOI or other citation details if detailed step-by-step protocols are available. |  | n/a |
|  |  |  |
| **Experimental study design (statistics details)** | **Yes (indicate where provided: page no/section/legend)** | **n/a** |
| State whether and how the following have been done**, or** if they were not carried out. |  |  |
| Sample size determination | We described the number of plants in each experiment in the manuscript. Detailed information can be found on Supplementary file (S1 Data.). The sample size for phenotype analyses was determined by maximum available number of plants in the same growth chamber at the same time. |  |
| Randomisation | No data were excluded from the analyses in this study | n/a |
| Blinding | We were aware of the allocation of samples in each experiment but we did not change any methods of data collection and analysis among sample groups. | n/a |
| Inclusion/exclusion criteria | No data were excluded from the analyses in this study. | n/a |
|  |  |  |
| Sample definition and in-laboratory replication | **Yes (indicate where provided: page no/section/legend)** | **n/a** |
| State number of times the experiment was replicated in laboratory | All experiments (Figure1-6) were conducted by more than 3 independent experiments. |  |
| Define whether data describe technical or biological replicates | More than three biologically independent experiments are conducted in Figure1-6.  We described the information in the figure legend or in Materials and methods as a subsection of Data analysis and statistics. |  |
|  |  |  |
| Ethics | **Yes (indicate where provided: page no/section/legend)** | **n/a** |
| Studies involving human participants: State details of authority granting ethics approval (IRB or equivalent committee(s), provide reference number for approval. |  | n/a |
| Studies involving experimental animals: State details of authority granting ethics approval (IRB or equivalent committee(s), provide reference number for approval. |  | n/a |
| Studies involving specimen and field samples: State if relevant permits obtained, provide details of authority approving study; if none were required, explain why. | We used only the experimental model organisms: *Marchantia Polymorpha and Oryza Sativa*. | n/a |
|  |  |  |
| Dual Use Research of Concern (DURC) | **Yes (indicate where provided: page no/section/legend)** | **n/a** |
| If study is subject to dual use research of concern, state the authority granting approval and reference number for the regulatory approval |  | n/a |

Analysis

| **Attrition** | **Yes (indicate where provided: page no/section/legend)** | **n/a** |
| --- | --- | --- |
| State if sample or data point from the analysis is excluded, and whether the criteria for exclusion were determined and specified in advance. |  | n/a |
|  |  |  |
| **Statistics** | **Yes (indicate where provided: page no/section/legend)** | **n/a** |
| Describe statistical tests used and justify choice of tests. | T-test was used to analyze whether two sample means are equal or not. This test was used in Figure3 and Supplementary figure 3. |  |
|  |  |  |
| **Data Availability** | **Yes (indicate where provided: page no/section/legend)** | **n/a** |
| State whether newly created datasets are available, including protocols for access or restriction on access. | All relevant data are within the paper and its Supporting Information files. |  |
| If data are publicly available, provide accession number in repository or DOI or URL. |  | n/a |
| If publicly available data are reused, provide accession number in repository or DOI or URL, where possible. |  | n/a |
|  |  |  |
| **Code Availability** | **Yes (indicate where provided: page no/section/legend)** | **n/a** |
| For all newly generated code and software essential for replicating the main findings of the study: |  |  |
| State whether the code or software is available. |  | n/a |
| If code is publicly available, provide accession number in repository, or DOI or URL. |  | n/a |

Reporting

| **Adherence to community standards** | **Yes (indicate where provided: page no/section/legend)** | **n/a** |
| --- | --- | --- |
| MDAR framework recommends adoption of discipline-specific guidelines, established and endorsed through community initiatives. Journals have their own policy about requiring specific guidelines and recommendations to complement MDAR. |  |  |
| State if relevant guidelines (eg., ICMJE, MIBBI, ARRIVE) have been followed, and whether a checklist (eg., CONSORT, PRISMA, ARRIVE) is provided with the manuscript. |  | n/a |
