# Supplementary material for: A conserved regulatory mechanism mediates the convergent evolution of plant shoot lateral organs
Source: PLoS Biol. 2019 Dec 9;17(12):e3000560. doi: 10.1371/journal.pbio.3000560 (PMC6901180; doi:10.1371/journal.pbio.3000560)
Supplement: Attachment — Submitted filename: NaramotoEtAl2019_PLOS-Bio_review.docx [file pbio.3000560.s011.docx]

This paper presents interesting and significant results on the regulation of lateral appendage development. These results have important implications for our understanding of the evolution of lateral appendages.

The paper is well written and illustrated. The methods and experimental design are appropriate and solid.

I see a minor issue with the statement on thylakoids (line 134): How many chloroplasts did you assess for that, i.e., what kind of numerical data support this statement about thylakoids?

The only other qualm I have, is with the section of the discussion on lines 320-324, concerning the two alternative hypotheses advanced by the authors regarding the evolution of lateral organs. Your first hypothesis is not specific enough and I don’t see how it would work. Here is why: if the ALOG gene controlled the development of lateral organs in the last common ancestor of the liverworts and the seed plants, where did this common ancestor have lateral organs: in the gametophyte, in the sporophyte, in both phases? The latter should have been the case, given that liverworts have lateral appendages in the gametophyte and seed plants have lateral appendages in the sporophyte. In this case we are, consequently, talking about a plant that had lateral appendages in both the gametophyte and the sporophyte phase and was the last common ancestor of liverworts and seed plants. This ancestor is the same as the last common ancestor of liverworts and tracheophytes (or polysporangiophytes). We don’t know what the earliest liverworts looked like, but it is likely that their gametophytes were thalloid (as suggested by their oldest known fossils) and could have had scales as seen in *Marchantia*. However, the oldest tracheophytes (and polysporangiophytes) had simple sporophyte axes devoid of lateral appendages. On the other hand, according to at least some phylogenies regarding relationships at the base of embryophytes (if not most of those phylogenies), the two ancestors you are talking about in the two hypotheses could have been the same. So, I think the two hypotheses need to be thoroughly reassessed and this part of the discussion needs to be re-written. Maybe the alternative hypotheses resulting from such a reassessment will have to do with whether the common ancestor had lateral appendages or not and in which phase of the life cycle (gametophyte or sporophyte) it had appendages. Furthermore, if ALOG function in lateral organ development was recruited independently in different embryophyte (land plant) lineages (lines 333-334), you may want to consider and speculate on what would have been their shared function (i.e. in some common ancestor) prior to these independent recruitment events.

Other than that, I only have a few terminology, grammar, and wording suggestions:

- Throughout the manuscript: check the grammar – I found some inadvertent plurals

- Line 31 and throughout – *Marchantia* is a formal genus name and it should italicized

- Lines 37-38 – the wording is a bit counterintuitive if you say that they specify the identity of lateral organs by repressing their growth. Please reword.

- Lines 59-60 – … forms a gametophyte axis that …

- Lines 69-70 – … and bilateral symmetry of the *Marchantia* scales resemble …

- Line 74 – is this, rather, Kenrick & Crane 1997? Does it not need to be a numbered reference?

- Line 116 – … much shorter than in the WT

- Lines 145-146 – “distance between each apical notch”??

- Lines 147-148, duplication of apical notches and axis separation – why not refer to this as bifurcation or, even better, just simply branching? After all, it is the same type of process as seen in the apical branching of tracheophyte sporophyte axes.

- Line 186 – the term merophyte has a more specific meaning than the general “cells derived from the apical cell”. The cells produced directly by division of the apical cell are designated as apical derivatives. A merophyte is a group of clonally related cells resulting from sequential cell divisions that originate in a single derivative of the apical cell of a meristem.

- Line 188 and elsewhere – triangular is a misleading descriptor of the shape of these cells, at least as illustrated in the figures referenced. Wedge-shaped may be a more accurate term.

- Line 253 – I have never encountered the term collar in describing archegonium parts. Do you mean archegonial neck?

- Lines 260-261 – your wording implies that a spikelet is a unit of a flower, which is inaccurate. Instead, the spikelet is made of one to several flowers. Please reword.

- Line 302 – your wording implies that rice and *Marchantia* are the earliest land plants. Please reword.

- Line 314 – Similarly, in tomato, *Sltmf* mutants …

- Line 374 – check wording
