# Supplementary material for: A conserved regulatory mechanism mediates the convergent evolution of plant shoot lateral organs
Source: PLoS Biol. 2019 Dec 9;17(12):e3000560. doi: 10.1371/journal.pbio.3000560 (PMC6901180; doi:10.1371/journal.pbio.3000560)
Supplement: Attachment — Submitted filename: Response to Reviewers_PLOSBiol20191012.docx [file pbio.3000560.s012.docx]

**REVIEWERS' COMMENTS:


Reviewer #1:
[identifies himself as Hiro-Yuki Hirano]

*Naramoto et al. revealed that the MpTAWAWA1 encoding an ALOG family protein is required for the development of lateral organs in Marchantia polymorpha, and that the function of ALOG genes to repress lateral organ growth is conserved in bryophytes and angiosperms. The authors identified the gene responsible for the morphological defects such as the formation of abnormal outgrowth and hyponasty of the thallus; the gene encoded a protein belonging to the ALOG family, some members of which are involved in organ and inflorescence development in rice. From the close examination of mutant phenotypes, the authors revealed that MpTAW1 is likely to regulate the development of lateral organs, such as ventral scales in the thallus and involucres in archegoniophore and to maintain the activity of the apical meristem in M. polymorph. Because MpTAW1 was expressed in the lateral organ primordia but not in the meristem, the authors concluded that this gene acts cell autonomously on the lateral organ development and non-cell autonomously on meristem activity. In addition, the authors showed that MpTAW1 rescued a mutant phenotype of the rice g1 mutant, suggesting that protein function of ALOG members is conserved in bryophyte and angiosperms. Thus, ALOG genes regulate the lateral organ development in the gametophyte in the bryophytes and in the sporophyte in the angiosperms. 

The results and the interpretation are interesting, and this work is likely to contribute the understanding of not only Marchantia development but also Evo-devo studies in plants. However, several concerns are raised, and I hope they are helpful to improve the work.*** 

>First of all, we would like to thank Prof. Hirano for providing us comments to improve our manuscript. In response to the comments, we have revised the manuscript with new data and would like to resubmit it for consideration by PLOS Biology. We do hope that you will find the revisions satisfactory. Our responses (blue) to the comments (**black**), along with added text (red) follow.

Major point:

***1. ‘MpTAW1 activity is required for….’,   line 142-199 
I am confused by the description of the results and interpretation in this section. The authors first described that the expansion of the region between the two apical notches (central lobe) is defective, resulting in the high density of the gemma cups. Then the authors examined the incorporation of EdU in the apical meristem to check the apical notch separation, that is, the expansion of the central lobe. 
I am wondering if the lobe expansion is a direct consequence of the activity of the apical meristem. The lobe is outside of the apical meristem consisting of the apical cell and merophytes. Thus, I think that the cells in the central lobe probably proliferate independent of the apical meristem activity. In Arabidopsis root, cells divide and proliferate in the division zone for root growth after differentiation of each cells such as epidermal and cortex cells originated from the root apical meristem. I think that the cell proliferation in the central lobe corresponds to that in the cell division zone of Arabidopsis root. 
Therefore, to uncover the cause of a reduction in central lobe growth in the Mptaw1 mutant, the authors should examine whether cell division is impaired in the central lobes during its growth. Conversely, if the cells in the central lobes are supplied only from the apical meristem, such data should be shown.*** 

>It is difficult to examine cell division activities in the central lobes during growth in Mp*taw1* mutants (which we have renamed to *los1* in our revised manuscript in response to comments – see below) because of the severe morphological defects in these mutants, which hamper our efforts to detect the central lobes. However, as suggested in the comment, we investigated the central lobes of wild type plants and found that they are mainly derived from the apical meristem, with very little cell division in this region. We performed EduU labelling during the growth in WT and we found that cell division activities within central lobes are much lower than in apical notch regions (in fact almost non-existent). Cell division activities are indeed high at the onset of the emergence of the central lobe, which initiates as two apical notches split; at this point the dividing cells that contribute to the central lobe still form part of the apical notches. We have added these data to the revised manuscript as Supplementary Figure 3D and 3E. From these analyses we conclude that cell division activity is low or absent in central lobes post emergence, and that the lobe-expansion defect seen in *los1* mutants is a consequence of defective meristem activity.

We have therefore added the following text to our manuscript:

“To determine whether the cell divisions that lead to the separation of the apical notches during branching occurs in the apical notches themselves or within the central lobes after they emerge, we measured EdU incorporation in these areas during WT development. We found that many cells incorporated EdU in the incipient central lobe as it forms in the apical notch region (S3 Fig D). In contrast, very little cell division is found within the central lobe once it has emerged (S3 Fig E). This indicates that cells in central lobes are mainly supplied from the apical notches, while the contribution of cell division activity within the central lobe for separating duplicated apical notches is low. Altogether, these data suggest that the rate of cell division in the apical notch is lower in the mutant than in WT, leading to an impaired separation of apical notches during branching.”

***2. Apart from the above comment, I agree that MpTAW1 is required for the activity of the apical meristem, as the authors showed lower incorporation of EdU. An additional examination such as the observation of division pattern of the apical cell in Mptaw1 would bring information that strengthen the authors’ statement in which MpTAW1 is required for the activity of apical meristem.***  

>To date there are no specific markers that label the apical cell in *Marchantia*, which makes it difficult to observe the detailed cell division patterns of the apical cell with confidence. While we could not directly follow the division patterns of the apical cell, we were able to observe the detailed cell structures around apical notches by labeling cell walls. We performed three-dimensional imaging by confocal laser scanning microscopy and visualized apical notch regions in different angles (vertical longitudinal, vertical transversal and horizontal sections around notches). These analyses, particularly the image obtained by horizontal sections, clearly showed defects in cellular structures and shapes in apical meristems, which we interpret as a consequence of defective apical cell divisions. These data further suggest that cell division pattern of apical cell is compromised in *los1* mutants. We have added these data to the revised manuscript as Figure S4A-4H, along with the following text:

“The WT apical meristem comprised a single wedge-shaped apical cell and surrounding merophytes (a group of clonally related cells resulting from sequential cell divisions in a single derivative of the apical cell of a meristem), in which the lateral merophytes and the apical cell display identical shapes (Fig 3H) [17]. In contrast, there were many wedge-shaped cells in *los1-1* mutants, in contrast to the single apical cell of wild type (Fig 3I; S4 Fig A-H). These defective cell division patterns in *los1* mutants lead to the formation of less strongly-invaginated notch regions (Fig 3I; S4 Fig A-H).”

*3. line 248-250*
***The authors stated that ‘loss of MpTAW1 function results in the homeotic transformation of involucres into more scale-like structures’, and ‘homeotic transformation’ is repeatedly used in DISCUSSION. However, there is little evidence that the abnormal involucres in the Mptaw1 mutant has ventral scale identity. To assert homeotic transformation, more close morphological examination should be added. Otherwise, I suggest that the authors weaken their claim by removing the word ‘homeotic transformation.***

>We agree with this suggestion. Accordingly, we did not use the word ‘homeotic transformation’ to explain the phenotype of *Marchantia* mutant. We have removed ‘homeotic’ from the text. The following is an example of our changes:

“This suggests that loss of LOS1 function results in the transformation of involucres into more scale-like structures.”

***4. The usage of the word ‘axis’
I am confused by the word ‘axis’. First, what does ‘axial systems’ (line 50, 52) mean? 
Second, it is also unclear the following words: ‘a bifurcating axis (line 143)’, ‘axis separation (line 148)’, ‘axis elongation (line 150)’, and ‘axis development (line 155)’. The authors should define these words more clearly. Some of the ‘axes’ should be drawn in the Figure 3A, if possible. But I think that the authors can describe this section more clearly (easily to understand) without the use of ‘axis’.*** 

> All land plants undergo apical growth driven by cell division activities of apical meristems and form a plant body along apical-basal axis. The word “axis” is commonly used to describe the morphology of the *Marchantia* thallus, for example by Solly et al. (2017). Therefore, we think it is appropriate to use the word axis, although we have tried to use other words when appropriate. Nevertheless, we have taken the comment into consideration and rewritten this section to make it easier for readers to understand. We define the word ‘axis’ and explain the phenomenon of ‘bifurcating axis’, ‘axis separation’, ‘axis elongation’, and ‘axis development’ in more detail:

“The WT thallus comprises an apical-basal axis produced by the activity of apical stem cells. The thallus undergoes periodic bifurcation and gemma cups develop along the midline of the dorsal surface. When the WT thallus bifurcates, a notch containing an apical stem cell forms on each of the two new apical-basal axes (Fig 3A). This process, the duplication of apical notches and the subsequent growth of thalli, is termed “branching”.”

Axes were also drawn into the Figure 3A as suggested.

***5. The TAW1, a rice ALOG gene, controls the activity of the inflorescence meristem. The authors claim that MpTAW1 is associated with the apical meristem activity. So, I would like to know whether MpTAW1 rescue the phenotype of loss-of-function mutant of TAW1.***

>As suggested, this is an important experiment. However, due to the redundancy of ALOG genes in rice, the loss of function mutant of OsTAW1 did not display a clear defective phenotype. Therefore, it is not easy to perform this experiment. We are trying to isolate rice mutants that carry mutations in multiple ALOG genes (e.g. double, triple mutants.) so that I can perform the suggested experiments in the future, but the timeline for the creation of these lines is beyond that which is possible within the period granted for resubmission.

***6. The ALOG genes in rice were already named in a preceding paper (Yoshida et al., 2009). But the authors used the different names such as TAW2 and TAW3 in Figure S1. The change of the gene names should be avoided, because it brings confusion. The name of MpTAW1 sound a little bit strange, because the function of MpTAW1 is similar to rice G1 rather than rice TAW1. If the authors want to avoid the use of MpG1, it would be good to create a new name from the mutant phenotype because this study started with the identification of morphological mutant.*** 

>We agree with this suggestion. Accordingly, we rewrote the names of rice ALOG genes, and they are now consistent with the preceding paper (Yoshida et al., 2009) (e.g. from “TAW2” to “G1L4”). We also renamed our *Marchantia* mutants as *lateral organ suppressor1* (*los1*) mutants.

***7. Transformation of rice g1 mutant.
What promoter did the authors use to express MpTAW1 in rice? There is no description about the experimental procedure concerning rice transformation in MATERIALS AND METHODS.
Are any effects other than the repression of sterile lemma growth observed in the transgenic g1 mutant overexpressing MpTAW1? Why the original lemma was not affected by MpTAW1 overexpression?*** 

>Thank you very much for pointing out this omission in the Materials and Methods. We used the 35S promoter to express LOS1 in rice *g1* mutants. We have now added this information to the Materials and Methods:

“For overexpression of *LOS1* in rice *g1* mutants, a plasmid carrying *pro35S::LOS1* was constructed. The *LOS1* genomic coding sequence was amplified by PCR and subcloned into pENTR/D-TOPO, which was subsequently integrated into pGWB2 by a Gateway LR reaction [48].”

Expression of *LOS1* driven by the 35S promoter did not induce any other effects than repression of sterile lemma growth. The growth of ordinary lemmas was not suppressed by the LOS1 in our hands. One possibility is that the sterile lemma is more sensitive to the expression levels of LOS1, which leads to the specific effect on the sterile lemma.

Minor points:

***1. RESULTS should be written in past tense.*** 

>In accordance with your suggestion, we rewrote the results in the past tense.

***2. line 136: ‘a higher photosynthetic activity in this tissue’
Is it possible to lead this discussion from only starch accumulation?***
> We agree that we do not have sufficient data to lead this conclusion. We therefore deleted the text’ Starch, not seen in WT scales, accumulates in the green outgrowth in Mp*taw1-1* mutants, suggesting a higher photosynthetic activity in this tissue (Figure 2L and 2M)’.

***3. ‘Molecular function of ALOG proteins (line 258, 267, 373)’ should be ‘protein function of ALOGs’, because there is no molecular analysis in the experiment using rice.*** 
> In accordance with your suggestion, we rewrote it as protein function of ALOGs.

***4. line 312, ‘The rice homolog…the sterile lemmas’ 
The reference should be cited.*** 
> In accordance with your suggestion, we cited Yoshida et al. (2009).

***5. Figure 2E: 
The organ with arrows appears to be out of focus a little.*** 
>This image is created by 3D reconstruction from multiple 2D images (xy plane) obtained by light sheet microscopy. Due to the positions of the lateral organs, the image has a lower resolution – the resolution is decreased by increasing the length between objectives and samples. Resolution also differs between xy plane and xz or yz plane. Therefore, this image is not out of focus, but suffers from a lower resolution because of technical limitations. We have tried to get better images but this remains our best image obtained so far. Therefore, we would like to keep this image but reduce the size of figures to reduce the impression of the decreased resolution. If this remains an issue, another possibility may be providing the data of Supplementary Figure 2A and 2B as main figures.

**6. Figures 4A-C:
Are these images taken from the ventral side? If so, please indicate this**.
>Yes, this image is taken from the ventral side. We indicate this information within the Figure legend.


Reviewer #2:
[also please see attached word doc for formatted version]

***This paper presents interesting and significant results on the regulation of lateral appendage development.  These results have important implications for our understanding of the evolution of lateral appendages.
The paper is well written and illustrated.  The methods and experimental design are appropriate and solid.***

>First of all, we would like to thank you for the positive evaluation of our work and detailed comments to improve our manuscript. In response to all the comments, we revised the manuscript with new data. Our responses (blue) to the comments (**black**), along with added text (red) follow.

***I see a minor issue with the statement on thylakoids (line 134): How many chloroplasts did you assess for that, i.e., what kind of numerical data support this statement about thylakoids?***

>In accordance with your suggestion, we added the information about the number of samples which we analyzed. We analyzed three independent gemmalings for both WT and mutants. We analyzed 97 chloroplasts in WT and 262 chloroplasts in mutants in total. We also provided compound images that were made up of 25-30 individual images on Supplementary Figure 2I and 2J so as readers to see the variations of chloroplasts among cells.

***The only other qualm I have, is with the section of the discussion on lines 320-324, concerning the two alternative hypotheses advanced by the authors regarding the evolution of lateral organs.  Your first hypothesis is not specific enough and I don’t see how it would work.  Here is why: if the ALOG gene controlled the development of lateral organs in the last common ancestor of the liverworts and the seed plants, where did this common ancestor have lateral organs: in the gametophyte, in the sporophyte, in both phases?  The latter should have been the case, given that liverworts have lateral appendages in the gametophyte and seed plants have lateral appendages in the sporophyte.  In this case we are, consequently, talking about a plant that had lateral appendages in both the gametophyte and the sporophyte phase and was the last common ancestor of liverworts and seed plants.  This ancestor is the same as the last common ancestor of liverworts and tracheophytes (or
polysporangiophytes).  We don’t know what the earliest liverworts looked like, but it is likely that their gametophytes were thalloid (as suggested by their oldest known fossils) and could have had scales as seen in Marchantia.  However, the oldest tracheophytes (and polysporangiophytes) had simple sporophyte axes devoid of lateral appendages.  On the other hand, according to at least some phylogenies regarding relationships at the base of embryophytes (if not most of those phylogenies), the two ancestors you are talking about in the two hypotheses could have been the same.  So, I think the two hypotheses need to be thoroughly reassessed and this part of the discussion needs to be re-written.  Maybe the alternative hypotheses resulting from such a reassessment will have to do with whether the common ancestor had lateral appendages or not and in which phase of the life cycle (gametophyte or sporophyte) it had appendages.  Furthermore, if ALOG function in lateral organ
development was recruited independently in different embryophyte (land plant) lineages (lines 333-334), you may want to consider and speculate on what would have been their shared function (i.e. in some common ancestor) prior to these independent recruitment events.***

>In accordance with your suggestions, we rewrote the discussion. Based on your suggestions, we discuss whether the common ancestor had lateral appendage or not and in which phase of the life cycle. We also discussed what would have been their shared function if ALOG function in lateral organ development was recruited independently in different land plant lineages. The new text is as follows:

“According to the first hypothesis, the common ancestor of liverworts and the seed plants developed lateral organs whose growth was controlled by the *ALOG* gene. Since lateral organs are present in the gametophyte and the sporophyte phase in liverworts and seed plants, respectively, under this scenario the common ancestor may have had lateral organs in both the gametophyte and the sporophyte phase. These structures subsequently diverged morphologically during the course of land plant evolution. However, this hypothesis is not well supported by fossil record. The earliest polysporangiophyte and tracheophyte fossils we know of, such as *Aglaophyton* and *Cooksonia*, possessed simple axes devoid of lateral organs [20, 21]. This suggests that their last common ancestor with the liverworts likewise lacked lateral organs.

An alternative hypothesis, which is in better accord with the fossil record, is therefore that the common ancestor of liverworts and the seed plants lacked lateral organs in the gametophyte and sporophyte phase but possessed an ALOG-mediated mechanism for controlling some other developmental process. The ALOG-dependent growth repression mechanism was subsequently recruited independently during the evolution of lateral organs in the separate lineages leading to the liverworts and seed plants. If the last common ancestor did not develop lateral organs, and since ALOG function regulates lateral organ development in both liverworts and seed plants, we suggest that ALOG function was recruited independently during the evolution of lateral organs in different lineages of land plants. The original ALOG function prior to these independent recruitment events may have been to control some aspect of apical meristem activities, since growth by apical meristems is a shared characteristic of land plants [38] and this ALOG function is found in both liverworts and seed plants. The recruitment of ALOG function during the independent evolution of lateral organs thus provides a molecular mechanism for the convergent evolution of growth repression in lateral organs”

Other than that, I only have a few terminology, grammar, and wording suggestions:

***- Throughout the manuscript: check the grammar – I found some inadvertent plurals***

>We checked the grammar, we hope we have corrected all usages of singular and plural.

***- Line 31 and throughout – Marchantia is a formal genus name and it should italicized***

>We have italicized *Marchantia* throughout.

***- Lines 37-38 – the wording is a bit counterintuitive if you say that they specify the identity of lateral organs by repressing their growth.  Please reword.***

>We reworded to: “ALOG genes regulate the development of lateral organs in both gametophyte and sporophyte shoots by repressing cell divisions.”

***- Lines 59-60 – … forms a gametophyte axis that …***

>We rewrote it according to your suggestions.

***- Lines 69-70 – … and bilateral symmetry of the Marchantia scales resemble …***

>We rewrote it according to your suggestions.

***- Line 74 – is this, rather, Kenrick & Crane 1997?  Does it not need to be a numbered reference?***

>Thank you very much for pointing out our mistake. Yes, this should be Kenrick & Crane 1997. We included this as reference.

***- Line 116 – … much shorter than in the WT***

>We rewrote it according to your suggestions.

***- Lines 145-146 – “distance between each apical notch”??***

> We removed the text ”The distance between apical notches increases along with the forward growth of thalli” since we realized that this text is unnecessary.

***- Lines 147-148, duplication of apical notches and axis separation – why not refer to this as bifurcation or, even better, just simply branching? After all, it is the same type of process as seen in the apical branching of tracheophyte sporophyte axes.***

>In accordance with your suggestion we used the word “branching”.

***- Line 186 – the term merophyte has a more specific meaning than the general “cells derived from the apical cell”.  The cells produced directly by division of the apical cell are designated as apical derivatives.  A merophyte is a group of clonally related cells resulting from sequential cell divisions that originate in a single derivative of the apical cell of a meristem.***

> We rewrote it according to your suggestions.

***- Line 188 and elsewhere – triangular is a misleading descriptor of the shape of these cells, at least as illustrated in the figures referenced.  Wedge-shaped may be a more accurate term.***

> In accordance with your suggestions, we reword it as “wedge-shaped.”

***- Line 253 – I have never encountered the term collar in describing archegonium parts.  Do you mean archegonial neck?***

> The term “collar” means a small sheath surrounding the base of unfertilized archegonium (Please see Shimamura 2016). The collar is composed by a ring of cells in single cell layer thickness. This structure develops into the pseudoperianth (protective structures of sporophyte) after fertilization of egg. We cited this reference (Shimamura 2016) at the first time the term “collar” appears in the text.

***- Lines 260-261 – your wording implies that a spikelet is a unit of a flower, which is inaccurate.  Instead, the spikelet is made of one to several flowers.  Please reword.***

> In accordance with your suggestions, we reword it as “flower clusters in grass species, basic unit of inflorescence, consisting of one or more flowers.”

***- Line 302 – your wording implies that rice and Marchantia are the earliest land plants.  Please reword.***

> We agree, and so removed the description ”the earliest land plants”.

***- Line 314 – Similarly, in tomato, Sltmf mutants …***

>We rewrote it according to your suggestions.

***- Line 374 – check wording***

>We rewrote it properly.
