# Supplementary material for: A conserved regulatory mechanism mediates the convergent evolution of plant shoot lateral organs
Source: PLoS Biol. 2019 Dec 9;17(12):e3000560. doi: 10.1371/journal.pbio.3000560 (PMC6901180; doi:10.1371/journal.pbio.3000560)
Supplement: Attachment — Submitted filename: SNVJ_Response to Reviewers_PLOSBiol20191030.docx [file pbio.3000560.s013.docx]

**REVIEWERS' COMMENTS:


Reviewer #1:
[identifies himself as Hiro-Yuki Hirano]

The authors have addressed most of my concerns and revised the manuscript.
Before publication, however, the authors should check more carefully the manuscript to remove inappropriate expression and grammatical errors. In addition, consistency should be maintained throughout the manuscript; eg., the usage of the mutant name as described below. I suggest the manuscript should be checked by a native speaker of English who preferably understand biology. Probably due to the error when the manuscript was converted to PDF file, figure legends are inserted inappropriate positions in the text.** 

>First of all, we would like to thank Prof. Hirano for providing us comments to improve our manuscript. In response to the comments, we have revised the manuscript with some corrections and would like to resubmit it for consideration by PLOS Biology. We do hope that you will find the revisions satisfactory. Our responses (blue) to the comments (**black**) follow.

Major point:
**- There are a number of inconsistent usages of mutant names: los1 mutant, los1-1 mutant, or los1 mutants, including the presence or absence of definite article (the). If the authors used one allele of los1 for phenotypic analysis, the name should be used consistently. Otherwise, respective allele names should be used.**

>Thank you very much for pointing out our omissions. We have now carefully checked our descriptions with the aid of a native English speaker. We have corrected the grammar and the inconsistency of mutant names. We believe we have corrected all the grammatical errors.

**Reviewer #2:
[identifies himself as Alexandru M.F. Tomescu]**

**I am happy with the revisions you have included.**

>We would like to thank Prof. Alexandru M.F. Tomescu for the positive evaluation of our work. Owing to your comments, the quality of our manuscript has improved very much. We would like to thank you again.
